# The Influence of Depression in the Evolution of Parkinson’s Disease: A Psychiatric Perspective

**DOI:** 10.1192/j.eurpsy.2024.629

**Published:** 2024-08-27

**Authors:** A. H. I. Abu Shehab, T. Simona, I. A. Ciureanu, D. C. Voinescu, L. Burlea, A. Ciubară

**Affiliations:** ^1^Psychiatry Department, “Elisabeta Doamna” Psychiatric Hospital, Galati; ^2^Psychiatry Department, Carol Davila University of Medicine and Pharmacy, Bucharest; ^3^Director of Communication Center, University of Medicine and Pharmacy “Grigore T. Popa”, Iași; ^4^Rheumatology department, “Dunărea de Jos” University - Faculty of Medicine and Pharmacy, Galati; ^5^Technology, Prothesis and Implantology, University of Medicine and Pharmacy “Grigore T. Popa”, Iași; ^6^Psychiatry Department, “Dunărea de Jos” University - Faculty of Medicine and Pharmacy, Galati, Romania

## Abstract

**Introduction:**

Parkinson’s disease (PD) is a neurodegenerative condition that is predominantly characterised by its motor symptoms. Nevertheless, it is important to note that non-motor symptoms, particularly depression often occur concurrently, exerting a substantial influence on the progression of the disease and the overall well-being of individuals affected by it.

**Objectives:**

The objective of this study is to examine the influence of depression on the advancement of Parkinson’s disease (PD) from a psychiatric perspective. This analysis will involve an assessment of the common neurobiological pathways involved and the potential implications for clinical treatment and care.

**Methods:**

A comprehensive assessment of the literature was conducted, focusing on clinical observations, neurochemical interactions, and neuroimaging investigations that provide insight into the concurrent presence of depression and Parkinson’s disease (PD). This study aimed to investigate the potential impact of depression on the severity of Parkinson’s disease symptoms, the course of the disease, and the responsiveness to treatment.

**Results:**

Depression in Parkinson’s disease (PD) is not only a reactive occurrence, but rather it may be attributed to common pathophysiological mechanisms, such as changes in dopamine and serotonin pathways. The coexistence of depression among individuals with Parkinson’s disease (PD) has been linked to heightened severity of motor and cognitive symptoms, accelerated development of the disease, and diminished effectiveness of therapy interventions. Furthermore, the presence of depression in individuals with Parkinson’s disease intensifies the psychosocial difficulties experienced by both patients and their carers.

**Conclusions:**

The recognition and management of depression in individuals with Parkinson’s disease (PD) is of utmost importance in order to enhance treatment approaches and enhance the overall well-being of patients. The establishment of interdisciplinary collaboration between neurologists and psychiatrists is necessary in order to guarantee a holistic approach to patient care.

**Disclosure of Interest:**

None Declared

